# Copaifera Officinalis

**Published:** 1866-08

**Authors:** Joseph Bates


*Copaifera Officinalis.*—(*Officinal Copaiba Tree.*) By JOSEPH BATES, M. D.

*Natural Order.*—*Fabaceæ*, Jussieu; or *Amyridaceæ*, Lindley.

*Description.*— Copaifera officinalis, in the Linnean artificial classification, belongs to class Decandria, order Monogynia.

*Generic Characters.*—Calyx, has four oblong, acute, spreading, concave sepals, sub-united base, within tomentose. The petals are wanting. (By some, the calyx is taken for the corol.) Legume ovate, one seeded. The copaifera officinalis is a large, elegant tree, with numerous branches near the top, and covered with a dense foliage. Leaves, alternate, large, primate, composed of from two to five pairs of ovate, entire, obtusely accuminate leaflets, from two to three inches in length, smooth, pellucidly punctate, somewhat shining, petioles short. Flowers white, subsessile, axillary, stamens filiform, incurved, exsert, anthers oblong.

*History.*—The genus Copaifera furnishes several species, in different localities, that supply the shops with the oleo-resin copaiba.

These trees are indigenous in various parts of South America, and in the West Indies. Brazil yields the best quality. The juice is obtained by making deep incisions into the trunks of trees, during the wet season, or immediately after. The juice flows freely, being clear and transparent, of an amber color, and about the consistence of olive oil. Its odor is aromatic, and at first rather pleasant; the taste is hot, bitter and nauseous. It is insoluble in water, though it imparts to its odor. Ether, alcohol, oils, and strong alkaline solutions are its solvents. It forms with alkalies a saponaceous compound which is insoluble in water. With one-sixteenth of its weight of magnesia, it forms a solid mass which is used to form pills.

Its constituents are mainly a volatile oil, and a resin which yields copaivic acid. More than one-half of its weight is essential oil. Its specific gravity varies from 950 to 1,000.

There was little or nothing known of this agent, so far as its history unfolds, previous to Piso's day. He furnished the earliest account of copaiba in 1648. This author claims that it cures gonorrhœa, leucorrhœa, and also diarrhœa; he mentions the use of copaiba in the cure of the first named disease by injections.

Marcgrave, who wrote at the same period, speaks of its virtues in dysentery, and other intestinal fluxes.

Morton (1720) included it among the balsamac medicines proper in consumption. From his time to that of Hunter, this medicine seems to have been more used for the treatment of bronchial affections than of gonorrhœa, but at the present day it is universally prescribed for the latter disease, and is regarded in the light of a specific for its cure. Even Hunter was far from appreciating its value, for while admitting that it lessens the disposition of the parts to form matter, he asserts that "not having at the same time the power of lessening the imflammation, it can be of little service."—(Stille.)

Copaiba, especially in the European markets, is often adulterated with oil of turpentine, or fixed oils.

*Action.*—In treating of its action, I shall avail myself of the remarks of Dr. Stille. "The operation of copaiba, according to Ricord, who has most completely investigated the subject, takes place upon the stomach, the bowels, the urinary apparatus, and the skin, and in some rare cases upon the nurvous centres.* Upon the stomach it shows its action by gradually destroying the appetite, exciting eructations with the characteristic smell and taste of the drug, or else nausea, retching, and even vomiting, with gastric

*A Treatise on the Venereal Diseases, by John Hunter, &c., Am. ed., 1853.

distress. When it disagrees with the stomach, it is apt at the same time to purge. Upon the urinary organs its influence is more uniform and more evident. It somewhat augments the quantity of this secretion and imparts to it a deeper color, a bitter taste, and a peculiar smell, which is somewhat fragrant. On standing, its surface is apt to be covered with an iridescent pellicle, and it becomes turbid from the presence of poisonous matter, which may be distinguished from albumen by its not readily subsiding, as well as its not answering to the tests for the latter substance. When the dose has been unduly large, it excites some heat and tenesmus in urinating, as well as a frequent desire to pass water, and even hæmaturia, and, at the same time, a feverish excitement of the whole system, fullness and frequency of the pulse, headache and thirst."

Copaiba is evidently absorbed into the general circulation ; this is implied by the peculiar odor which is imparted to the breath, by its modification of the urine, by its action upon the skin, and by its influence upon bronchial inflammations.

The substances which it most closely resembles, both in composition and properties, are the turpentines. Copaiba is said to be somewhat of a stimulant, diuretic, laxative, and in large doses frequently an active purgative. It sometimes occasions an eruption upon the skin, resembling that of measels, and accompanied with an itching, and tingling sensation. Ricord maintained that when it occasioned cutaneous eruptions, its effects on the urinary passages were very slight. These cutaneous eruptions were first observed by Montegre, who described certain red spots as appearing on the skin whenever the urethral discharges were suspended.

Delpech noticed a miliary eruption connected with gastric disturbance.—(Stille.)

Armstrong also observed itching and an eruption of the skin produced by copaiba. In 1828, Dr. Hewson, of Philadelphia, published a notice of several such cases. In two of these, the eruptions resembled uticaria, and in two others it had the characters of roseola. Mr. Judd has also described "a puniceous mottled eruption," and one of maculæ, and Desruells observed uticaria proceeding from this cause. These eruptions are most apt to manifest themselves when the medicine does not agree with the stomach, and they disclose the probability that the disease is not favorably influenced by it. Ricord remarks that it is not uncommon to see patients in whom the running, after having been dried up, reappears along with the cutaneous eruption.

Copaiba sometimes creates alarming symptoms, by its effects on the nervous system. Ricord saw one patient, in whom temporary hemiplegia was produced, in consequence of taking too large doses, which ceased upon the occurrence of a rubeolar eruption; and in another, an attack of convulsions was terminated in like manner.

Dr. Roquette, of Nantes, believes that copaiba only acts as a curative agent, in affections of the urino-genital organs, by means of the peculiar qualities which it imparts to the urine, and that it is useless in inflammations of these portions of the genital mucous membrane which do not come in contact with the urine during its passage. As it has been satisfactorily proved, that when copaiba is tolerated, it is eliminated from the system almost exclusively by the kidneys, and that consequently it is present in large quantity in the urine. Dr. Roquette considers, that when we administer this remedy, we should endeavor to prevent diarrhœa, vomiting, nausea, etc., as much as possible, in order that the remedy may solely leave the economy through the kidneys.—(Bost. Med. and Surg. Jour., vol. 56, p. 128.

Oil of copaiba acts very much as copaiba itself, but less powerfully.—(Stille.)

USES—DYSENTERY.—Armstrong was in favor of administering copaiba in protracted and obstinate cases of dysentery, as a means of healing the ulcerated condition of the intestines. He remarks that it was formerly used for this purpose, and that we have been perhaps too hasty in discarding it. Dr. La Roche, as quoted by Stille, has likewise recommended it in the declining stage of acute dysentery, when the stools are mucous rather than bloody. Dr. Hatheway, of Illinois, is very decided in relation to the utility of this agent, in the disease under consideration. He alludes to copaiba as a specific in dysentery, and is inclined to regard it as such in all stages of the disease.

At a meeting of the Medical Society of London, Mr. Roberts related a case of nephritis, in which, after bleeding, and the ordinary treatment of that disease, some inflammatory symptoms still remaining, and suppression of urine more particularly, he exhibited copaiba in ten drop doses three times a day, with the effect of restoring the secretion.—(Lancet, June 27, 1846, p. 705.)

*Chronic Gastro-Enteritis.*—Mr. Robarts has reported it to be very effectual in this disease, when given in doses of from seven to ten minims three times a day, made into an emulsion.—(Stille.)

From its reputed efficacy in diseases of mucous membranes, there is sufficient presumptive evidence that this drug will be found highly useful in many diseases of the alimentary canal.

*Gonorrhœa.*—The use of copaiba in this disease appears to have been introduced by the natives of South America, and the knowledge thus obtained introduced into Europe. During the last century, Hunter and Swediaur brought it into more general notoriety. Accidents frequently teach important lessons in medicine. In 1804 a patient of Ribes, of Paris, took by mistake an ounce of copaiba at a single dose. He was purged, suffered colic and loss of appetite, but the gonorrhœa he labored under was cured.

Drs. Delpech and Ribes gave copaiba on the outset of this disease, however great the inflammation might be; and they affirm having obtained the greatest advantages from its early employment. Dr. Velpeau, in order to obviate the inconveniences of this remedy when taken in large doses, proposed to administer it in glysters, and numerous successful experiments leave no doubt whatever of the efficacy of this method.

Whenever this method has been had recourse to, it has been more successful, where a laxative has been previously exhibited. After the bowels have been evacuated, and are in a quiet state, then the injection of copaiba should be administered, and the results are the same, as if taken into the stomach. The oil of copaiba was introduced into the French practice by Mr. Dublane, Jr., in the treatment of gonorrhœa. Drs. Bard and Cuellerier witnessed its happy results in thirty patients whom they cured in five or six days of this disease.

It has since been administered by several physicians with complete success. Chopart's mixture is a very good combination, which is prepared with mint-water, alcohol, syrup, āā ℥ ij; spirit of nitric ether, ℥ j; orange flower water, ℨ ij M. S. Two tablespoonfuls in the morning, one at noon, and one at night; and continue for twelve days. Ansiaux adopted the plan of using this mixture in 1812, from the very beginning of the attack, and found that it frequently produced a speedy cure.

Armstrong, in 1818, adopted this method on the suggestion of Dr. Dawson, who had for several years been accustomed to its use. Armstrong began the use of the remedy in the very first stage of virulent gonorrhœa, and came to the conclusion, from his own experience, that it would not fail once in twenty times to arrest completely the progress of the disease, if given in sufficient doses, and continued several days after the disappearance of the discharge.

Subsequently, Delpech (1822) directed, that after reducing the inflammation, if necessary, by local and general depletion, a drachm of copaiba should be administered morning and evening, gradually increasing the dose to twice this quantity three times a day, and giving opium if it purged. Ricord advocates the same treatment, and increases the dose if necessary, and can be tolerated to one ounce in twenty-four hours.

Clarus, in Germany, adopted a more cautious method, beginning with smaller doses of a weak emulsion, and subsequently administering it pure in doses of from twenty to forty drops three times a day.

It is very important to commence the treatment during the forming stage of the attack ; it is then more readily arrested. The remedy should be continued, and gradually diminished, for several days subsequent to the arrest of the discharge.

Mr. J. Hilton recommends first, in gonorrhœa, acetate of potash in half drachm doses every four hours, and he remarks, even where the disease has not yielded, it has been repeatedly found, that after a few doses of copaiba the cure has been completed, and a relapse prevented.—(Braith. part 22, p. 375.)

Copaiba produces in the morbid mucous membrane over which it passes, mixed with urine, an action incompatible with the diseased one; "hence its mode of action seems to be that of substitution." —(Stille.)

Mr. Dallas, of Odessa, used several times a day injections of the following emulsion: ℞. Copaiba, ʒ v; yolk of egg, No. 1; extract of opium, gr. j; water, ℥ vij; and is stated to have employed it, without any other remedy, with complete success.

*Psoriasis.*—Numerous authors could be instanced who have lauded copaiba in the treatment of cutaneous affections. (The following is from the Boston *Medical and Surgical Journal*, vol. 10, p. 271.)

"The internal administration of arsenic, and the topical application of the oil of juniper and of tar, in the treatment of psoriasis, enjoy a certain reputation as is well known among dermatologists. But the local medication is but too often merely palliative, and even arsenic does not always ensure the patient against a return of the disease, while its administration, however prudently directed, is far from being always free from inconvenience. These considerations induced M. Hardy to try other means, and his choice fell upon the internal employment of balsam of copaiba. The following details concerning the treatment now in use in the service of this physician, at the Hospital of St. Louis, are taken from the *Bulletin de Therapeutique.*

"M. Hardy generally commences the treatment in the dose of about three-fourths of a fluid drachm, which he subsequently increases to a drachm, and a drachm and a half. It is given in the morning before eating, and in the intervals of the meals, during the day. It is continued for a considerable time, at least a month, and sometimes longer. It is generally combined with local treatment, but it is sometimes employed successfully alone.

Copaiba thus administered generally causes diarrhœa, which is, however, well borne by the patients, and does not ordinarily prevent them from taking food, even with appetite.

"It rarely gives place to erythema, sometimes produced by this drug. The scaly eruption generally gets well in all places at the same time, and the improvement is not always more marked, at the beginning, in the inferior extremities, as occurs in other modes of treatment. It first shows itself in the parts most lightly attacked, and from thence spreads towards the places of election. When the scales become detached, the sujacent skin is generally sound, though sometimes still a little red. Psoriasis existing in patches becomes converted into psoriasis circinata, the healing beginning at the centre of the patch; and the psoriasis circinata is transformed into P.— guttata."

The following summary of facts, taken from the thesis of M. Paul Dupuy, one of the pupils of M. Hardy, will enable the reader to appreciate the effects of this treatment, and judge how much benefit we can hope to obtain from it:

"A patient who entered the hospital two or three months since for psoriasis, and who was treated by the ordinary remedies (arsenical preparations, baths, ointments, etc.), still retained a small patch of the disease on the left shoulder. On the 12th of February, about a fluid drachm of copaiba was substituted for the arsenical solution; in one week the patch had almost disappeared, and at the end of three weeks the patient was cured.

"A second patient, aged forty-nine years, several members of whose family had been affected with skin disease, had, at the age of twenty-one, an eruption of psoriasis, which disappeared spontaneously at the end of a few months. Afterwards the disease reappeared from time to time; but since 1840, the psoriasis had constantly persisted, sometimes in one place, sometimes in another. At his entrance into the hospital, in November, 1855, he had patches of psoriasis on the elbows, knees, loins, scalp, and ears. He was treated by the arsenical solution, vapor, sulphur and alkaline baths, and ointments containing oil of juniper, and afterwards proto-iodide of mercury; but his psoriasis still remained, on the 28th of February following, in the form of large patches on the elbows, knees and loins—though the scales had diminished in quantity. At this time he was placed upon the use of copaiba, in the dose of a drachm gradually increased to double that quantity. The local treatment was continued, but the arsenic was omitted. At the end of a week there was a perceptible improvement, and an the 25th of March the patient left the Hospital perfectly cured, having continued the copaiba up to that time. He was seen again on the 11th of June, but presented no symptoms of relapse. In the third patient, the affection dated from five weeks only. It consisted in psoriasis in patches and guttata, having its seat on the elbows, arms, fore-arms, legs and thighs, and was accompanied by severe itching during the night. He entered the Hospital on the 8th of March, and two days afterwards was put upon the electuary of copaiba, in the dose of a fluid drachm at first, gradually increased to a drachm and a half. On the 15th there was a sensible improvement; the guttæ were smaller, and in several places had in a great part disappeared. There were fewer scales, and there was hardly any itching. On the 3d of April there was hardly any of the disease left on the thighs, arms, and posterior part of the leg. In other places the eruption was less prominent and less scaly. Ou the 15th, general subsidence of the patches in those places from which the scales had become detached. On the 13th of May there was no psoriasis except on the places of election, and the front of the legs. June 1st, the left arm and leg are well; 15th, the right elbow is completely cured; there remain only a few scales on the right knee. On the 20th, the patient was discharged at his own request. During the whole course of the treatment, the digestive functions were regularly performed, and the stools were not more frequent than usual."

*Vesical Catarrh.*—Many authors, such as Hoffman, Bretonneau, Barbier, Delpech, and Dr. LaRoche, have found copaiba very serviceable in the treatment of this affection. According to Dr. LaRoche, moderate doses are the most successful, because they agree better with the stomach and can be continued for a longer time. He also advised that the medicine should be occasionally suspended, in order to allow the stomach an opportunity of recruiting, and to prevent the risk of too constant an irritation of the affected parts.—(Stille.)

Others employed injections of copaiba into the bladder in these cases, to the extent of two ounces, with a like quantity of barley water, as it would seem, successfully.

*Bronchial Affections.*—This medicine, in bronchial affections, has had its advocates from the earliest period of its history. Many writers have recommended it in the treatment of consumption. Fothergill (1769) pointed out certain cases, in which it would be injurious, such as are attended with bloody and purulent expectoration, referring thereby to cases of pulmonary tubercles. Hoffman and Monroe speak highly of its benefits in chronic pulmonary affections. Halle used this agent in the cure of chronic bronchitis. Armstrong (1818) regarded it somewhat in the light of a specific, for chronic inflammation of the mucous membrane of the trachea and bronchia. He administered it in doses, varying from thirty to eighty drops, three times a day. A favorite prescription of of Morgagni for chronic affections of the lungs, was copaiba in combination with sulphur. This combination was commended by Armstrong. Dr. LaRoche, of Philadelphia, demonstrated the great advantages of copaiba in chronic inflammation of the lungs in 1827. For the same purpose it was highly prized by Belling-hiere and Bertini, of Turin. Many physicians in this country hold that copaiba in chronic bronchitis, unattended with fever, has greater claims upon the confidence of the profession than any other remedy.

*Leucorrhœa.*—Copaiba, alternated with quinine and iron, three times a day, will often be found serviceable.

*Local Application.*—Formerly copaiba was much used as a dressing for wounds and sores. Sacks recommended it highly as an application to the sore nipples of nursing women. He is confident, if the remedy is applied before there is any loss of substance, it will arrest the evil.

*Chilblains.*—Dr. Ruschenberger recommends its application in chilblains as an excellent remedy.

*Administration.*—It may be given pure, or on sugar, or floated upon water. The dose generally may be from twenty to sixty drops; sometimes gradually increased to one ounce in twenty-four hours. A very good form in which to take it, is that of capsules; or it may be taken in the form of pill with magnesia.

— The theory that ozone and antozone are two opposite electrical states of oxygen, and that ordinary oxygen is composed of the two opponents balancing each other, has led to the theory that all matter is in this condition, and several important observations have been made which go to sustain the view.

